# Understanding Health Literacy among University Health Science Students of Different Nationalities

**DOI:** 10.3390/ijerph191811758

**Published:** 2022-09-17

**Authors:** Henrietta Bánfai-Csonka, Bálint Bánfai, Sára Jeges, József Betlehem

**Affiliations:** 1 Doctoral School of Health Sciences, Faculty of Health Sciences, University of Pécs, 7621 Pécs, Hungary; 2 Institute of Emergency Care and Pedagogy of Health, Faculty of Health Sciences, University of Pécs, 7621 Pécs, Hungary; 3 Emergency Department, Clinical Center, University of Pécs, 7624 Pécs, Hungary

**Keywords:** health sciences university students, subjective health literacy level, functional health literacy level, NVS, HLS-EU-Q16

## Abstract

An adequate level of health literacy is essential for clear communication between patients and health care workers. The internationalization of universities is increasing in the field of health care. The aims of our research were to measure (1) the level of health literacy and its correlation among university students and (2) the relationship between the different instruments measuring health literacy. A cross-sectional study was conducted in the 2020/2021 academic year. The questionnaire included questions on sociodemographic status, study data, health status, and health literacy level. According to the HLS-EU-Q16 health literacy questionnaire, more than half of the students had a limited HL level in disease prevention (52.4%) and health promotion (58.4%) subindexes. Nationality was found to be an influencing factor (*p* < 0.001). According to the NVS, 80.1% of the students had an adequate HL level. A significant correlation was found between the results and nationality (*p* = 0.005). None of the Chew questions demonstrated a correlation with nationality (q1 *p* = 0.269, q2 *p* = 0.368, q3 *p* = 0.528). Nationality is a key factor in the level of subjective and functional health literacy. We need to measure both types of levels to see the real results.

## 1. Introduction

Internationalization in higher education is a current trend worldwide. Studying abroad brings different nationalities and cultures together, but it is a real challenge to understand and accept other social norms, ways of communication, and symbols [1,2].

Immigration is a negative predictor when we measure health literacy (HL) level [3]. Being an immigrant has a socially determining role in terms of access to health care, health outcomes, health status, and health literacy [3,4,5,6]. Those who cannot speak the national language in a country often have a limited health literacy level [5,7]. The cultural background also has a deep impact on the level of HL [8].

Defining health literacy is not an easy task. In the last 30 years the definition has changed a lot. One of the first and most popular definitions was provided by the World Health Organization (WHO) [9].

Sorensen et al. created an integrated model of health literacy. According to that definition, health literacy requires four competences: obtaining, understanding, processing, and applying information [10]. To measure the level of health literacy, several instruments have been created [11,12,13], some of which provide subjective information, while others provide objective or functional measurements. These tools involve questions related to the health status, access to information, and tasks that assess basic literacy and numeracy.

Multiple factors can influence the level of health literacy. There are studies that have tested the relationship between socio-economic data (gender, age, level of education, and general household income) [3,14,15], health behavior (smoking habits, regular eating, physical activity, and alcohol drinking) [16,17], and the level of health literacy. Being a woman [17], an elderly person [3,17], or a non-smoker [17] positively influences the health literacy level.

We found several studies that focused on general population HL [12,14,16,18,19,20]; however, we found fewer data about the health literacy level of medical/health sciences professionals and students [17,21,22,23,24,25].

The importance of studying the attitude of health care professionals must be emphasized, as it will contribute to appropriate communication and relationships between the patient and the medical professionals and health care workers (in many cases, patients expect guidance from the professionals) [26]. Furthermore, such insights are indispensable for finding an effective method for training and education.

In an earlier study, participants in the Care-Health-Pedagogy program, as well as those who demonstrated higher levels of health literacy, assessed their own health condition more positively than their peers [16].

Research studies have reported that students in a higher years of study have better health literacy level compared with those in the first year of study [17,24]. According to a study conducted on students of medicine and social care in Spain and France [21], student nurses scored the highest in the subindex of the health care system, while 36.5% of the respondents reached the “sufficient” level.

The level of health literacy among students appears to be inadequate [21,23,24,27]. In Hungary, similar research was conducted only among students from different faculties of health sciences, and it displayed equally poor results [22]. A study from Nepal revealed that most medical students have only a moderate health literacy level. Furthermore, medical university students had higher scores than those who were taking part in training courses only [23]. In Turkey, the results for student nurses suggest a correlation of higher health literacy with secondary education, the financial situation of the family, and the level acquired on the Health Literacy European Survey (HLS-EU) scale [24]. Other research demonstrated that the higher the number of health pedagogy courses a student has completed, the higher the level of health literacy they reach regarding their access to and comprehension of information in the questionnaire’s subcategory of health improvement [15]. Health literacy demonstrates a relationship not only with a person’s sociodemographic and educational background, but also with one’s mental well-being [28].

A strong correlation was found between higher standards of living and physical, mental, social, and teenage well-being. Health literacy correlates negatively three mental disorders—stress reactions, signs of depression, and impulsiveness—while it positively influences the standards of living [29]. Improving the health literacy level from a young age (as early as primary school) seems to be a crucial factor in improving societies’ well-being [30]. Including health literacy in curriculum is an essential stage, because it is not only about the knowledge, but also developing behavior habits [31]). The educators of young children were found to have a higher level of health literacy compared to that of the general Hungarian population [32].

The improved results of pharmacy students who had completed a health literacy program testify that development is possible, even as late as the university level [25]. An 8 h training session targeting medical professionals from three countries (Italy, Netherlands, and North Ireland) involved research on health literacy, tasks in communication, decision making, and enforcement. The level of health awareness improved immediately after the training and continued to show improvement after 6–12 weeks [33]. The notion of HL can be found in many places, so there are many definitions and interpretations. Different methods are available to assess its level. It would be important to develop a uniform interpretation, definition, and related tools [16].

In the Hungarian language we can also find some measurement tools to evaluate the subjective and objective level of HL. For measuring the subjective level of HL, the HLS-EU-Q47 [34] and the BHLS [35] was validated in general population. The shorter version of HLS-EU-Q47, the HLS-EU-Q1,6 was only used in previous studies but it was not really validated [36]. For measuring the objective level of HL, the NVS [35] and S-TOFHLA [37] also have validated with the prefilter Chew questions [37].

The aims of our study were to measure the level of subjective and functional health literacy and their correlations among Hungarian and foreign health sciences university students. An additional aim was to measure the relationships between the different instruments of health literacy. We sought answers to the following questions:

- What is the level of subjective and functional health literacy for undergraduate students in the faculty of health sciences?
- Are there any differences between Hungarian and foreign students’ health literacy level?
- Is it possible to identify any relationship among the measuring instruments?

## 2. Materials and Methods

We conducted a cross-sectional study, which was measured with a self-created questionnaire. It contained non-standardized items (data on sociodemographic and educational factors) and standardized sets of questions to assess health literacy and physical activity. The Health Literacy European Survey 16-item questionnaire (HLS-EU-Q16), Newest Vital Signe (NVS) and Chew questions were used. For Hungarian students, the questionnaire was written in Hungarian, and for foreign students, it was available in the original English language.

### 2.1. Sample

The survey was conducted among students of various fields of health sciences from various academic years studying in English or Hungarian at the Faculty of Health Sciences, University of Pécs. Respondents included both Hungarian and foreign students who had an active status in the 2020/2021 academic year during the spring semester. Questionnaires that were inappropriately filled out were excluded. Before answering the survey, the students read an informational sheet regarding the research and then completed a statement of consent, agreeing to take part in the survey.

### 2.2. Data Survey

The questionnaire that included the informational sheet and the consent statement was sent to the students online through the NEPTUN educational administration system between January and May 2021. Those students who agreed received the link to the form via their private e-mail address.

After registering to Office 365 Microsoft Forms, the questionnaire was answered online as a Forms document. The survey began with questions related to sociodemographic data and educational background. After these initial questions, the subjective aspect of health literacy was explored with the help of the HLS-EU-Q16 questionnaire, which examines the compound level of health literacy and its three subdimensions (health care (HC), disease prevention (DP), and health promotion (HP)). Four other subindexes can also be created: obtaining, understanding, appraising, and applying for information [38]. According to the scoring of the questionnaire, we have to standardize the index points from 0 to 50. The four groups of HL levels were created according to these points (0–25: inadequate; 26–33: problematic; 34–42: sufficient; 43–50: excellent) [38]. This questionnaire was not validated in Hungarian language but is was used in an earlier study as well (Kun).

This section followed the Chew questions [39] and then the Newest Vital Signe (NVS) questionnaire [40]. These measurement tools were validated before in Hungarian language among the general population and parents [35,37]. The Chew questions includes three questions to measure the level of health literacy in three different domains: problems learning: “How often do you have problems learning about your medical condition because of difficulty understanding written information?”; confidence with forms: “How confident are you filling out medical forms by yourself?”; and help with reading: “How often do you have someone help you read hospital materials?” [39]. The NVS measures the level of functional HL with the help of a product description on a box of ice cream. It contains six questions for understanding what the participants read and their counting skills. If the patient answered the first four questions correctly, they had an adequate HL level. They get 1 point for each correct answer. The scoring was as follows: 0–1 point is the likelihood limited HL level, 2–3 points is the possibility of limited HL level, and 4–6 points is the adequate HL level [40].

### 2.3. Statistical Analysis

Statistical analysis was carried out using the SPSS 24.0 program. We calculated the Cronbach alpha to see the consistency of the questionnaire. Analyzing internal consistency among the questions (Cronbach-alpha test) in HLS-EU-Q16 showed the following results in the different dimensions (health-care system: 0.776, prevention: 0.725, health promotion: 0.789, cHL: 0.880). This means that in all dimensions, the questions are cohesive.

We conducted descriptive statistical analyses (mean, SD) and mathematical statistics to examine relationships between variables. Categorical variables were compared using Khi-square test or Fisher’s exact test, as appropriate (e.g., HL level and sociodemographic characteristics). ANOVA was used to evaluate the association between age and sociodemographic characteristics. The significance level was defined when *p* < 0.05.

## 3. Results

### 3.1. Characteristics of the Sample (Sociodemographic, Educational, Health Status, and Health Behavior)

Data on sociodemographic, education, health status, and health behavior are displayed in Table 1.

Female (86.13%) and Hungarian (86.1%) respondents are overrepresented in our sample. The mean age of the respondents was 21.86 (SD = 4.6) years. The proportions of urban and rural residents are almost equal. Most of them have an average (59.2%) or higher (27.7%) economic status.

We had participants from all grades (first (40.45%), second (28.83%), third (17.23%), and fourth (13.49%)) and all specializations in Bachelor’s degrees. Most of them did not have to postpone their studies.

If we assess Hungarian and foreign students and the data on sociodemographic background and education, we can conclude that there is a relationship between economic status (*p* < 0.001), place of residence (*p* = 0.001), whether the participant’s mother (*p* < 0.001) and father (*p* = 0.004) have a permanent job, whether they have postponed their studies (*p* < 0.001), and course specialization (*p* < 0.001) and nationality.

The body mass index (BMI) of the students was 23.05 (SD = 4.26), which can be considered in the normal range. Most of the students (81.1%) reported no chronic disease, and 74.5% of the participants reported having never smoked. The majority (70.6%) reported not having a general practitioner in the location where they studied. More than three-quarters of the respondents (77.2%) evaluated their own health condition as ‘good’ or ‘very good’. Most of the participants (72.7%) had heard the term health literacy, mostly during their studies or through various forms of media. Only 9.7% of the participants thought that the level of their health literacy is limited.

The subjective evaluation of the participants’ self-reported health and their BMI classification demonstrated a significant relationship (*p* = 0.002). Those who have a better self-reported health are more likely to have normal BMI. Overall, 165 students who have a normal BMI index said that she/he have a good or very good health condition. However, no relationship was established among smoking habits (*p* = 0.415), the presence of a chronic disease (*p* = 0.081), and their self-assessed level of health literacy (*p* = 0.282). Similarly, whether they had a private physician at the location of their study demonstrated no connection with having a chronic disease (*p* = 0.151) or with the subjective evaluation of their health literacy (*p* = 0.265) or health status (*p* = 0.553).

### 3.2. The Analysis of Health Literacy

#### 3.2.1. The Results of the HLS-EU-Q16 Questionnaire

The study showed that participants have limited health literacy level regarding obtaining (38.9%), understanding, processing (53.6%), and applying (58.4%) information (Table 2).

Nationality was found to be a potential influencing factor regarding only the application of information (*p* < 0.001). Foreign students were better at applying information than Hungarian students. A total of 61.24% of Hungarian students and 40.53% of foreign students were in the limited category in this subindex.

For the sociodemographic data, subjective economic status could be an influencing factor in information search (*p* = 0.041) and application (*p* = 0.049), while education proved to be significant regarding information processing (*p* = 0.040), and whether the participant’s mother had a permanent job was influential regarding comprehension (*p* = 0.027) and processing of information (*p* = 0.008). More information about the relationships between sociodemographics, study, and health behavior data and nationalities can be found in Table 1.

Concerning the data on education, academic year and the student’s course work schedule were not relevant in any category; however, postponement of studies had probably relevance in applying information (*p* = 0.031), and specialization was influential in obtaining (*p* < 0.001), understanding (*p* = 0.012), and processing (*p* = 0.004) information. Among those who did not postpone (80.1%), a higher proportion (49.5%) belonged to the category of limited health literacy than those who did (8.9%). Studying as a nurse seems to be an influencing factor, because in obtaining, understanding, and processing information they have a higher level of HL than the others.

Table 3 shows that 38.2% of the students demonstrated limited health literacy in the health care (HC) subindex, 52.4% in disease prevention (DP), and 58.4% in health promotion (HP). Nationality proved to be potential influential in all three indices (HC *p* = 0.029, DP *p* = 0.002, and HP *p* < 0.001). Other sociodemographic data did not show relationships concerning these subindexes. Specialization displayed relationship in HC (*p* = 0.016), postponement of studies in HP (*p* = 0.031), and student’s course work schedule in DP (*p* = 0.036).

#### 3.2.2. Results of the NVS Questionnaire

Table 4 shows how different nationalities scored in the same categories, together with the relationship of the data with the sociodemographic background and education.

The table demonstrates that 80.1% of the students testified to having an adequate level of functional HL on the scale. Nationality seems to be relevant here (Hungarian students scored higher) (*p* = 0.005), while other sociodemographic factors were not influential.

#### 3.2.3. Results of the Chew Questions

Table 5 shows the answers to the Chew questions displayed according to nationality. Most of the respondents often (50.9%) or always (31.8%) required help with understanding hospital documentation. However, understanding documents related to the participants’ own health only rarely (31.4%) or sometimes (31.1%) caused problems. Respondents never or rarely (65.2%) felt confident when filling in hospital documentation. None of the questions referenced demonstrated an association with nationality (q1 *p* = 0.269, q2 *p* = 0.368, q3 *p* = 0.528).

The results of the questionnaires focusing on HL conducted with differing academic levels were also apparently unrelated to nationality (whether a student is a Hungarian or a foreigner).

#### 3.2.4. Analyzing the Relationships between the Results of the Different Questionnaires

Comparing the results of the NVS and Chew questions, we can see that there is a association between the first two questions (q1 *p* = 0.027, q2 *p* = 0.041, q3 *p* = 0.294). Hence, data concerning understanding medical documentation show a positive relationship with the results of the survey on functional HL.

#### 3.2.5. Comparing the Results of the Questionnaires with the Questions Investigating Self-Assessed Health Literacy

In the case of the HLS-EUQ-16 questionnaire, when information processing was investigated, self-assessed health literacy showed a relationship with the results of the questionnaire in all categories (obtaining information *p* = 0.04, understanding *p* < 0.001, processing *p* < 0.001, and application *p* < 0.001). Additionally, there was a significant relation in all three subindexes between assessing one’s own health (HC *p* = 0.014, DP *p* = 0.011, and HP *p* = 0.018) and self-reported health literacy level (*p* < 0.001).

A connection between the NVS questionnaire and the self-assessed level of health literacy (*p* = 0.248) could not be established. Furthermore, whether one is familiar with the concept of health literacy proved uninfluential.

For the Chew questions, the results of all three questions correlated with the self-assessed level of health literacy (q1 *p* < 0.001, q2 *p* = 0.037, q3 *p* = 0.001); however, being familiar with the concept of health literacy proved relevant only in the case of the third question (q1 *p* = 0.063, q2 *p* = 0.249, q3 *p* = 0.04).

There was apparently no connection between assessing one’s own health and the presence of a chronic disease in either tool or questionnaire.

## 4. Discussion

In the present study, we assessed HL among health sciences students (Hungarian and foreign students) with different measurement tools (HLS-EU-Q16, NVS, and Chew questions) and compared them with sociodemographic, education, and self-reported health data. Most of the respondents were women, and the average age was 21.88 SD = 4.61. First year students were overrepresented in the survey. These sociodemographic data are characteristic of the students of the Faculty of Health Sciences, as currently most of the health care professionals are women in Hungary. It is also characteristic that the higher the grade, the lower the number of students, as an increasing number of them postpone or cancel their studies. In Hungary, the attrition number is very high in the universities, in Bachelor’s degrees it is 36–39%, in undivided training it is 32–36%, and in Master’s degrees it is 19–20% [41,42]. This explains the high number of first graders in the survey.

Among our respondents, nationality seems to be a possible factor for influencing HL level; however, other sociodemographic factors (self-assessed economic status, gender, age, education, whether one’s mother or father has a permanent job, and place of residence) had no significant result. They may have had an effect, but only a weak relationship could be established. This result contradicts other surveys on HL conducted among members of the general public [10,22,43]. Nationality (whether the student is Hungarian or a foreign student) can have an influential role on the level of HL comprehension on both subjective (HLS-EU-Q16) and functional (NVS) assessment scales. This is a good reason universities need to include the HL in the curriculum of the health sciences faculties. Because of the internationalization of the universities, the curriculum needs to focus on different nationalities and help students integration to the national health care system as a health care provider [1,2].

Based on the HLS-EU-Q16 questionnaire, more than 50% of the students demonstrated limited health literacy in all subindexes (HC, DP, HP), except the one concerning the health care system. In contrast, on the basis of the NVS survey investigating functional health literacy, 80.1% of the respondents demonstrated an adequate level of health literacy. These results are worse than those of surveys conducted in Hungary in 2016 [22].

On the basis of our survey, there was no apparent relation among factors concerning educational level and health literacy according to either of the questionnaires. Grade [22,24] proved to be influential in earlier studies, but our survey could not find any statistical connection. However, by examining the percentages, we can state that the higher one’s grade is, the higher one’s health literacy. According to our survey, specialization is an influencing factor. To study as a nurse seems to be a factor to have a better health literacy level. In an earlier study, nurses also showed a better HL level than others who study other health sciences [21].

In international studies, unhealthy behaviors (e.g., smoking) [16] and chronic diseases [44] are relevant for HL, contrary to our survey. The presence of a private physician at the place of study also appeared not to be relevant.

Our HLS-EU-Q16 data showed that the subjective assessment of health and health literacy proved to be determining factors in all subindexes, as opposed to an earlier study conducted among university students [22]. Those students who evaluated their health literacy higher and assessed their state of health as better demonstrated a higher level of functional health literacy. On the basis of the NVS, we cannot establish connections for any of the cases, while on the basis of the Chew questions, assessing one’s own health literacy showed a significant influence on all three questions.

## 5. Limitations

Some of the measurement tools for evaluating the level of health literacy were validated in Hungarian language earlier (Chew questions and NVS), but the HLS-EU-Q16 was not validated only the longer version HLS-EU-Q47. However, it was used before in Hungarian studies.

We conducted our survey during the COVID-19 pandemic, when only a few foreign students could apply to and begin their studies at our university. Consequently, the number of foreign students was significantly lower than that of Hungarian students. Furthermore, there is a disparity in the number of students in various specializations, as not all specializations are available in English for foreign students. Because of the consecutive sampling and not a randomized, we cannot make conclusions for all students who study at health sciences.

The respondents answered the questionnaire online from their homes; hence, it is beyond our control whether they responded independently on their own.

The corrections of these factors can be a good basis for new extended research in all Hungarian universities in which health professionals are trained.

## 6. Conclusions

In conclusion, nationality seems to be a key factor concerning both subjective and functional health literacy. Other sociodemographic factors did not influence the level of health literacy among the students in our study. Analyzing data about educational levels and postponing studies had an influence as opposed to grade and work schedule.

More than half of the students at the University of Pécs Faculty of Health Sciences testified to having limited health literacy in all subindexes of the HLS-EU-Q16 questionnaire. On the basis of the Chew questions, most of them indicated problems with understanding medical information. According to the NVS, most of the students demonstrated an adequate level of functional health literacy.

According to our results, we need to measure both types of levels to see the real results. There are significant differences between the Hungarian and foreign students’ HL level, but neither group falls into the excellent category. The level of health literacy needs to be improved among university students in the health sciences.

## Figures and Tables

**Table 1:** Summarizing sociodemographic, educational and health status, and health behavior data and relationships between the factors and nationality.

Characteristic	National	International	Total	*p* Value ^1^
**Sociodemographic data**				
**Gender**				<0.001
**male**	24	13	37 (13.87%)	
**female**	206	19	230 (86.13%)	
**Age**	21.97 ± 4.83	21.36 ± 2.8	21.88 ± 4.6	0.628
**Economic status (subjective)**				0.005
**very bad**	2	2	4 (1.5%)	
**bad**	21	10	31 (11.6%)	
**average**	135	23	158 (59.2%)	
**good**	67	2	69 (25.8%)	
**very good**	5	0	5 (1.9%)	
**Settlement**				0.001
**town**	117	17	134 (50.2%)	
**village**	65	3	68 (25.5%)	
**county town**	48	17	65 (24.3%)	
**Regular job (mother)**				<0.001
**yes**	203	21	224 (83.9%)	
**no**	27	16	43 (16.1%)	
**Regular job (father)**		24		0.004
**yes**	194	13	218 (81.6%)	
**no**	36		49 (18.4%)	
**Educational background**				
**Grade**				0.843
**1st**	95	13	108 (40.45%)	
**2nd**	64	13	77 (28.83%)	
**3rd**	40	6	46 (17.23%)	
**4th**	31	5	36 (13.49%)	
**Work schedule**				0.920
**day**	214	34	248 (92.89%)	
**correspondents training**	16	3	19 (7.11%)	
**Postponement**				<0.001
**yes, because of my studies**	6	4	10 (3.75%)	
**yes, because of other things**	12	30	43 (16.1%)	
**no**	211	3	214 (80.15%)	
**Specialization**				<0.001
**paramedic**	26	0	26 (9.74%)	
**nurse**	16	10	26 (9.74%)	
**nutrition**	44	11	55 (20.59%)	
**physiotherapy**	110	13	123 (46.07%)	
**recreation**	6	0	6 (2.25%)	
**midwife**	3	3	6 (2.25%)	
**health visitor**	10	0	10 (3.74%)	
**diagnostic and analytics**	15	0	15 (5.62%)	
**Health condition, health** **behavior**				
**GP in the town**				0.059
**yes**	49	14	63 (23.59%)	
**no**	181	23	204 (76.41%)	
**Health condition (subjective)**				0.002
**bad**	5	1	6 (2.25%)	
**average**	48	7	55 (20.6%)	
**good**	132	11	143 (53.56%)	
**very good**	45	18	63 (23.59%)	
**Heard about HL ^2^**				0.778
**yes**	166	27	194 (72.65%)	
**no**	64	9	73 (27.35%)	
**HL ^2^ level (subjective)**				0.957
**inadequate**	3	1	4 (1.5%)	
**problematic**	20	2	22 (8.24%)	
**sufficient**	178	28	206 (77.16%)	
**excellent**	29	6	35 (13.1%)	

^1^ Significant level *p* < 0.05. ^2^ HL: health literacy.

**Table 2:** Data on the various dimensions of health literacy.

Level of HL ^1^	Dimensions of HL ^1^ [n (%)]			
	Obtain Information	Understand Information	Process Information	Apply Information
**Inadequate**	19 (7.1%)	52 (19.5%)	52 (19.5%)	62 (23.2%)
**Problematic**	85 (31.8%)	91 (34.1%)	91 (34.1%)	94 (35.2%)
**Sufficient**	104 (39%)	78 (29.2%)	77 (28.8%)	62 (23.2%)
**Excellent**	59 (22.1%)	46 (17.2%)	47 (17.6%)	49 (18.4%)

^1^ HL: health literacy.

**Table 3:** The level of HL in different subindexes depending on the HLS-EU-Q16 questionnaire.

Level of HL ^1^	Subindexes of HL ^1^ [n (%)]		
	HC Subindex	DP Subindex	HP Subindex
**Inadequate**	17 (6.4%)	38 (14.2%)	62 (23.2%)
**Problematic**	85 (31.8%)	102 (38.2%)	94 (35.2%)
**Sufficient**	115 (43.1%)	73 (27.3%)	62 (23.2%)
**Excellent**	50 18.7%)	54 (20.2%)	49 (18.4%)

^1^ HL: health literacy.

**Table 4:** Relationships between NVS results, sociodemographic data, study data, and nationalities.

HL^1^ Level	National	International	Together	*p*-Value *
Limited (inadequate)	6	4	10 (3.7%)	
Possible to limited (limited)	33	10	43 (16.1%)	
Adequate	191	23	214 (80.1%)	
**Sociodemographic data**				
Nationality				0.005
Sex				0.937
Age				0.697
Economic status				0.067
Education level				0.424
Settlement				0.737
Regular job (mother)				0.154
Regular job (father)				0.112
**Study data**				
Grade				0.353
Work schedule				0.333
Postpone				<0.001
Specialty				0.094

^1^ HL: health literacy; *significant level: *p* < 0.05

**Table 5:** Relationship between the answers of Chew questions and nationalities.

Question	National	International	*p*-Value
**How often do you have someone (like a family member, friend, hospital/clinic worker, or caregiver) help you read hospital materials? (Help Read)**			0.269
Never	1	0	
Occasionally	10	2	
Sometimes	27	6	
Often	122	14	
Always	70	15	
**How often do you have problems learning about your medical condition because of difficulty understanding written information? (Problems Reading)**			0.368
Never	47	10	
Occasionally	76	8	
Sometimes	71	12	
Often	29	6	
Always	7	1	
**How confident are you filling out forms by yourself? (Confident with Forms)**			0.528
Never	46	10	
Occasionally	107	11	
Sometimes	57	12	
Often	14	3	
Always	6	1	

## Data Availability

The dataset supporting the conclusions of this article is available from the corresponding author on reasonable request.

## Abbreviations

AI	apply information
BMI	Body Mass Index
DP	Disease Prevention
HC	Health Care
HL	Health Literacy
HLS-EU-Q16	European Health Literacy Survey Questionnaire 16 item
HP	Health promotion
NVS	Newest Vital Signe
OI	obtain information
PI	process information
UI	understand information
WHO	World Health Organization
q1	first question
q2	second question
q3	third question
